# The Effect of Integrated Aerobic and Cognitive Training (Activ4Brain) on Cognition and Neurophysiology in Older Adults: Protocol for a Controlled Trial

**DOI:** 10.2196/92631

**Published:** 2026-09-17

**Authors:** Hicran Besikci, Kaan Akalp, Luís Rama, Beatriz Branquinho Gomes, José Pedro Leitão Ferreira, Susana Mouga, Inês Bernardino, Miguel Castelo-Branco, Ana Rita Sousa e Silva, Ana Maria Teixeira, Maria José Ribeiro

**Affiliations:** 1 CIBIT - Coimbra Institute for Biomedical Imaging and Translational Research Coimbra, Instituto de Ciências Nucleares Aplicadas à Saúde (ICNAS), University of Coimbra, Coimbra, Portugal; 2 Faculty of Sport Sciences and Physical Education, CIPER, University of Coimbra, Coimbra, Portugal; 3 Faculty of Psychology and Educational Sciences, University of Coimbra, Coimbra, Portugal; 4 Faculty of Medicine, University of Coimbra, Coimbra, Portugal; 5 CINEICC - Center for Research in Neuropsychology and Cognitive Behavioral Interventions, Faculty of Psychology and Educational Sciences, University of Coimbra, Coimbra, Portugal; 6 RISE-Health, Center for Translational Health and Medical Biotechnology (TBIO), School of Health (ESS), Polytechnic of Porto, Rua Dr. António Bernardino de Almeida, 400, Porto, 4200 - 072, Portugal, 351 915234593

**Keywords:** healthy aging, age-related cognitive decline, cognitive dysfunction, exercise, aerobic exercise, cognitive training, arousal, pupil-linked arousal, electroencephalography, pupil, inflammation, immune function biomarkers

## Abstract

**Background:**

There is a need for efficient and engaging strategies that will mitigate the effects of aging on cognitive and physical functions while keeping users motivated for long-term adherence.

**Objective:**

This study protocol is designed to investigate, in older adults, the advantage of integrating cognitive training with aerobic exercise (AE) in fun group sessions to potentiate the effect of training on cognition while improving physical function. The protocol explores the effects of a novel intervention, the Activ4Brain program, on cognition and physical function as well as the underlying neurobiological mechanisms.

**Methods:**

The program consists of group AE classes that include computerized games designed to train cognition during exercise, taking advantage of exercise-induced arousal. Cognitive training task difficulty is adapted throughout the intervention as users improve their performance. Importantly, the setup allows for simultaneous body movements to maintain aerobic activation. This is a 3-arm controlled trial, using a parallel design with a 1:1:1 allocation to an experimental group that is engaged in the Activ4Brain program (24 sessions, twice a week), an active control group that is engaged in AE sessions, and a passive control group. Participants are tested at baseline and after the intervention. The main study outcome is changes in cognition. Additional outcomes include changes in brain and autonomic function, levels of inflammation and neuroprotective markers, cardiovascular and physical functional fitness, and body composition.

**Results:**

Data collection started in January 2024 and finished in April 2025. Seventy-three individuals (40 women and 33 men) were enrolled in the study and assigned to the 3 groups. The average age was 66 (SD 6) years. Data analyses are ongoing, with the publication of the results estimated for 2026 and 2027.

**Conclusions:**

This study will produce knowledge important for the development of efficient strategies to fight age-related decline.

## Introduction

Cognitive training holds the potential to be a successful nonpharmacological treatment for age-related cognitive decline and dementia. However, there is a need for the design of better tools, as existing interventions show little to no benefit for preventing or delaying cognitive decline [1]. Similarly, physical exercise has been explored as an intervention for improving cognitive function. However, although in patients with mild cognitive impairment the cognitive effects of exercise are positive [2], in the general population the effects are small, suggesting caution in the use of exercise for prevention of age-related cognitive decline [3].

Despite these shortcomings, physical exercise can be used synergistically with cognitive training to boost their effects on cognition. Exercise increases arousal and potentiates neurogenesis and neuroplasticity through the release of brain-derived neurotrophic factor [4,5]. Thus, enhancing brain arousal before and during cognitive training might be beneficial because the brain will be more apt to attend to the information presented (enhanced arousal) and to learn and consolidate new skills (enhanced synaptic plasticity). Moreover, learning plays an important role in promoting neuroplasticity and the survival of neurons [6]. Therefore, adding cognitive challenges to periods of physical activity may maximize the benefits of exercise on cognition. Another reason why it is beneficial to include exercise in a program tackling cognitive decline is that exercise engages the noradrenergic system, one of the neural systems associated with the arousal response. Noradrenaline has a protective role against the onset and progression of cognitive decline [7-9], probably through its anti-inflammatory role [10]. Repeated activation of the noradrenergic system during regular exercise sessions might lead to beneficial permanent changes in the structure, connectivity, and function of the noradrenergic system, leading to improved brain function and brain health.

Taking into consideration the synergy between physical activity and brain function, we designed the program Activ4Brain as a nonpharmacological strategy to reduce the impact of age-related cognitive decline. Previous studies have explored the use of simultaneous physical exercise and cognitive training [11]. Exergames are commonly used in this type of intervention; however, the difficulty to adapt to the needs and abilities of the study participants limits their capability to address specific cognitive difficulties [12-15]. Other studies use single cognitive tasks, thereby training a very limited set of cognitive skills [16], or use designs that do not adapt task difficulty as the participants improve their performance during the intervention, an important feature to optimize learning [17,18]. Our novel program was designed to address these limitations. The Activ4Brain program consists of group aerobic exercise (AE) classes that include simultaneous computerized tasks designed taking into consideration the cognitive domains that respond to training and are more affected by aging, and the best practices for inducing long-lasting brain and behavioral plasticity [19,20]. During AE classes, visual stimuli are displayed on a screen at the front of the room, and the participants are instructed to respond according to the cognitive training tasks’ rules using foot pedals, while standing and moving their bodies to maintain arousal levels throughout. In this way, this program integrates cognitive training in AE. Task accuracy is automatically recorded, and the tasks’ difficulty levels are adjusted weekly to ensure participants are challenged by the cognitive training tasks throughout the intervention. The program consists of 24 sessions, held twice a week. Each session is 50 minutes long, during which 5-minute bouts of AE (used to increase arousal levels) alternate with 5-minute bouts of cognitive training. The program Activ4Brain is designed to be applied in group classes in a dynamic and fun manner at the sound of music, promoting social interaction (an important factor for successful aging), increasing its user motivation and long-term adherence, and facilitating its application on a large scale.

This study protocol was designed to assess the cognitive impact of the Activ4Brain program and to elucidate the neural and biochemical mechanisms underlying its effects. Participants are tested at baseline and after the 3-month intervention. Participants are assessed for cognitive function using a comprehensive cognitive assessment protocol including memory and executive function tests. This is our main outcome. To examine brain and autonomic function, we acquire the electroencephalogram (EEG), the pupilogram, and the electrocardiogram (ECG) during rest and while participants are engaged in a value-based learning task (reinforcement learning task). Reinforcement learning is a skill that is impaired in older people [21], involves the engagement of the arousal system [22], and depends on executive function [23]. We hypothesize that training executive function will result in improvements in reinforcement learning, thereby asserting transfer learning to untrained tasks. These analyses will elucidate if the Activ4Brain program induces changes in brain and autonomic activity during rest and cognitive task performance and behavioral improvements in untrained tasks (far transfer) and will facilitate the investigation of the neurobiological mechanisms that underlie the cognitive changes. To investigate if the relationship between exercise and improved cognition is linked to a reduction in inflammation and an enhancement in neuroprotection, we also measure inflammatory and neuroprotective blood markers. To examine the program’s impact on physical health markers, we assess body composition, physical functionality, and physical and cardiovascular fitness.

## Methods

### Trial Registration and Protocol Timing

As a behavioral, nonpharmacological intervention aimed at improving cognitive health and well-being in aging, prospective registration was not initially anticipated by the authors, and the study was registered on ClinicalTrials.gov (NCT07108413) only after completion of data collection. Data collection started in January 2024 and finished in April 2025. The protocol registration was submitted in July 2025 and published in August 2025. The aim of this retrospective trial registration and publication is to document this extensive protocol in detail, including a novel intervention. This will allow the protocol to be reproduced, and it will assist with the reporting of the results that will be divided into several publications focused on the analyses of the different outcomes and different aspects of the intervention.

No public involvement is planned for any stage of the study, including in the protocol design.

### Study Design and Intervention Protocol

**Figure 1.**
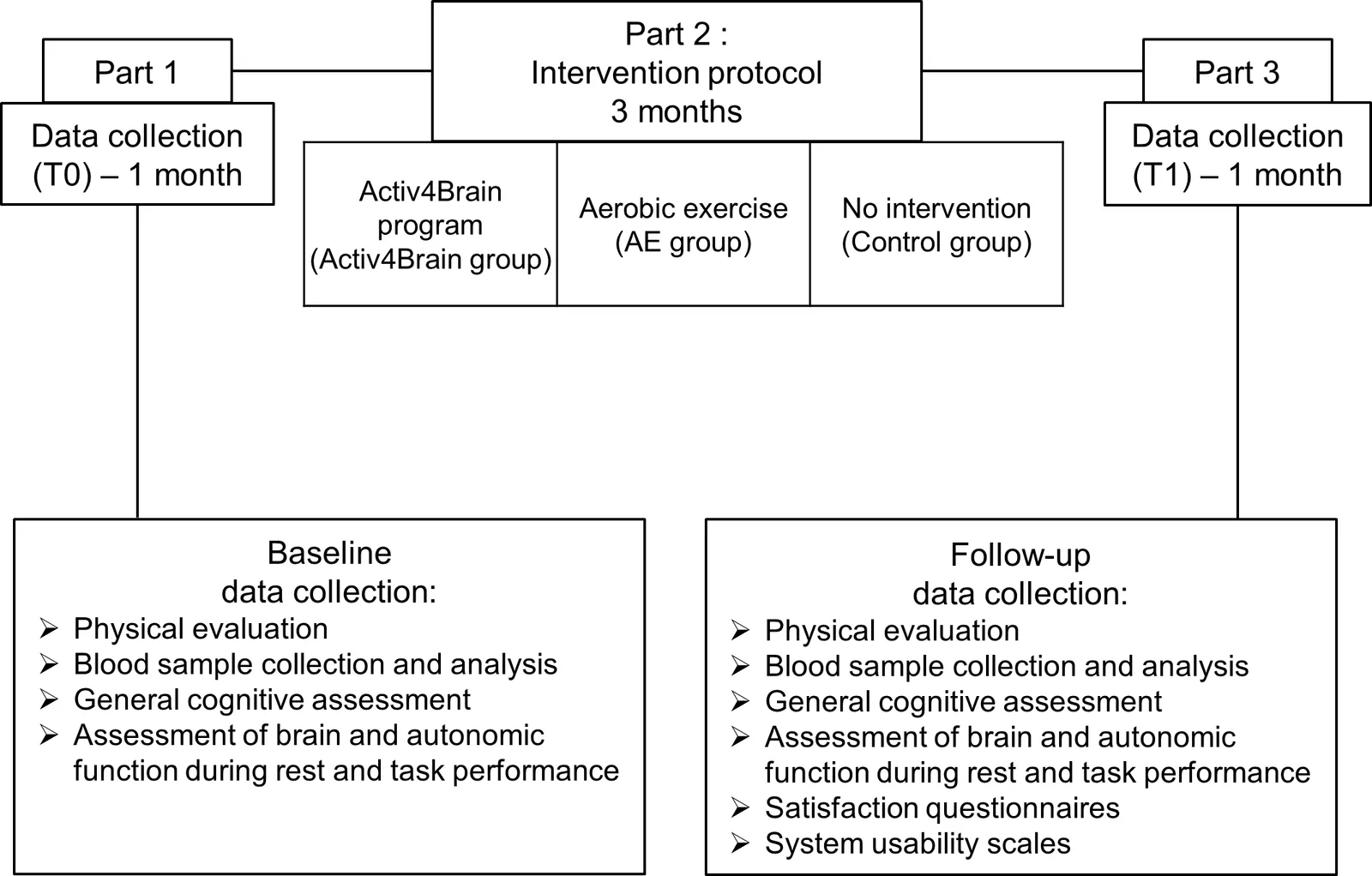
Study flowchart. The research protocol is divided into 3 main parts. Part 1, with a duration of 1 month, corresponds to the initial data collection phase (T0), serving as the baseline assessment. Part 2 corresponds to the 3-month intervention protocol, which includes the Activ4Brain program (Activ4Brain group) and the aerobic exercise-only program (AE group). Part 3, with a duration of 1 month, corresponds to the follow-up data collection phase (T1) conducted after the intervention.

Our study consists of a 3-arm controlled trial with a parallel study design involving cognitively healthy older people. Participants are allocated into the Activ4Brain group and 2 control groups: an active control group engaged in AE alone and a passive control group, instructed to maintain their daily routines. The passive control group is included to control for the effect of test-retest. The AE group is included to assess if including cognitive training leads to a greater effect on cognition than AE alone and to control for the participants’ motivation to engage in the evaluation sessions, which might be decreased in passive control groups. The group allocation ratio is 1:1:1. The intervention consists of 24 sessions, held twice a week for 3 months. The participants are tested at baseline and after the 3-month training programs (Figure 1).

### Ethical Considerations

The study protocol was approved by the Faculty Ethical Committee of the Faculty of Sports Science and Physical Education of the University of Coimbra with the reference number CE/FCDEF-UC/00082023. All participants are asked to read and sign a consent form. This protocol is reported in accordance with the SPIRIT (Standard Protocol Items: Recommendations for Interventional Trials) 2025 guidance (see Checklist 1 for a completed checklist). The results will be reported following the CONSORT (Consolidated Standards of Reporting Trials) extension for nonpharmacological interventions [24].

### Participant Recruitment, Eligibility Criteria, and Sample Size

Participants are recruited from Portugal’s Coimbra District through personal communications, press releases, social media posts, and distribution of informative leaflets and posters. Recruited participants receive comprehensive information regarding data collection procedures, exercise protocols, and measures of protection of personal information procedures. Participants who volunteer to participate in the study are interviewed for eligibility and asked to sign an informed consent form.

Inclusion criteria are cognitively healthy men and women between 55 and 75 years of age, with a physical condition compatible with the practice of moderate AE. Exclusion criteria include any disability or health problem incompatible with moderate AE and objective cognitive impairment. The Physical Activity Readiness Questionnaire is used to screen contraindications for physical exercise. Before the intervention, we ensure participants’ fall risk is low using the Timed Up and Go (TUG) test [25].

Participants are instructed to continue any ongoing concomitant care. Details of the concomitant care received are recorded at baseline and, to assess any changes, at follow-up.

Sample size determination has been made using the G*power (version 3.1.9.7; written by Franz Faul, Universität Kiel, Germany) program. As reported in previous studies [26,27], we expect a small to moderate effect size of the Activ4Brain program on cognitive function (our primary end point). We calculated a sample size of 66 participants by setting Type I error (α) at 0.05, Type II error rate (1−β) at 0.95, and an effect size η^2^ of 0.06 (corresponding to an effect size *f* of 0.25, using the option for effect size specification as in G*Power 3.0) for repeated-measures ANOVA, within-between interaction, with 3 groups and 2 repeated measures. Accepting a dropout rate of 10%, 75 participants are recruited.

### Randomization and Blinding

This trial is a quasi-randomized controlled unblinded trial. After recruitment, participants are assigned to 1 of 3 groups based on sex and age, ensuring that each group has similar sex ratios and age ranges. These balanced groups are then randomly assigned, using the random permutation of integers function from MATLAB (The MathWorks Company Ltd), to 1 of the 3 interventions: Activ4Brain, AE, or control.

### Details of the AEs Programs

The AE and Activ4Brain groups engage in 50-minute exercise sessions of moderate intensity (60%‐80% of maximal heart rate), including a 5-minute warm-up, 40 minutes of AEs, and a 5-minute cool-down. The warm-up period includes similar body movements as the main part of the exercise and dynamic stretching, while the cool-down period consists of static stretching. The main part of the exercise session consists of 4 blocks (Table 1). The sessions of the AE group are composed of a sequence of the four 5-minute blocks of AE repeated twice. In the Activ4Brain group sessions, the blocks consist of 5-minute AEs and 5 minutes of cognitive training (Table 2) and are performed only once per session. The Activ4Brain group performs exercise that includes the same rhythmic movements matched in intensity range and duration to the exercise performed by the AE group. The Activ4Brain participants are instructed to keep moving, marching in place, while performing the cognitive training tasks to maintain the increased heart rate. Each group class is composed of 4 participants with a narrow age range to ensure similar physical and cognitive performance.

**Table 1.** Example of the main body structure of a session for the aerobic exercise group^a^.

Block	Rhythmic body movements	Duration (min)
1	V step Marching in place Step jack	5
2	Arms and knees up Marching in place Box step	5
3	High knee touches (with bounce) Marching in place Bounce sky punch	5
4	Four steps forward and return with back walk Marching in place Sidesteps (2 right-2 left)	5

a The sequence of the 4 blocks, 1 to 4, is repeated twice in the same order to make a total of 40 minutes of exercise.

**Table 2.** Example of the main body structure of a session of the Activ4Brain group, including aerobic exercise and cognitive training.

Block	Activity	Description	Duration (min)
1	Rhythmic body movements	V step Marching in place Step jack	5
1	Cognitive training	Task 1 while marching in place	5
2	Rhythmic body movements	Arms and knees up Marching in place Box step	5
2	Cognitive training	Task 2 while marching in place	5
3	Rhythmic body movements	High knee touches (with bounce) Marching in place Bounce sky punch	5
3	Cognitive training	Task 3 while marching in place	5
4	Rhythmic body movements	Four steps forward, return with back walk Marching in place Sidesteps (2 right-2 left)	5
4	Cognitive training	Task 4 while marching in place	5

In both training groups, music with a tempo of 130 to 135 beats per minute-32 count is used to regulate rhythm and enhance motivation [28]. The heart rate of every participant is monitored during the exercise classes with the Polar Team Pro System (Polar Team Pro; Polar Electro), which enables the simultaneous tracking of multiple participants and the monitoring of the exercise intensity in real time, ensuring adequate exercise intensity across all groups.

### Cognitive Training Tasks

The Activ4Brain program includes cognitive training tasks targeting 5 cognitive domains: processing speed, inhibitory control, selective attention, working memory, and decision-making. To maintain participants’ motivation and ensure that the tasks are challenging as participants improve their skills, the tasks’ difficulty level is increased if the class average accuracy for the previous week exceeds 80%. The cognitive training tasks were designed using Psychophysics Toolbox Version 3 [29,30] in MATLAB. In each session, 4 cognitive domains are trained in blocks of 5 minutes each (Table 2). At the end of each week, participants are sent by email their average performance metrics for that week, consisting of the accuracy in each cognitive training task, median reaction time, and reaction time variability (SD), with a written explanation of the meaning of each measure.

**Figure 2.**
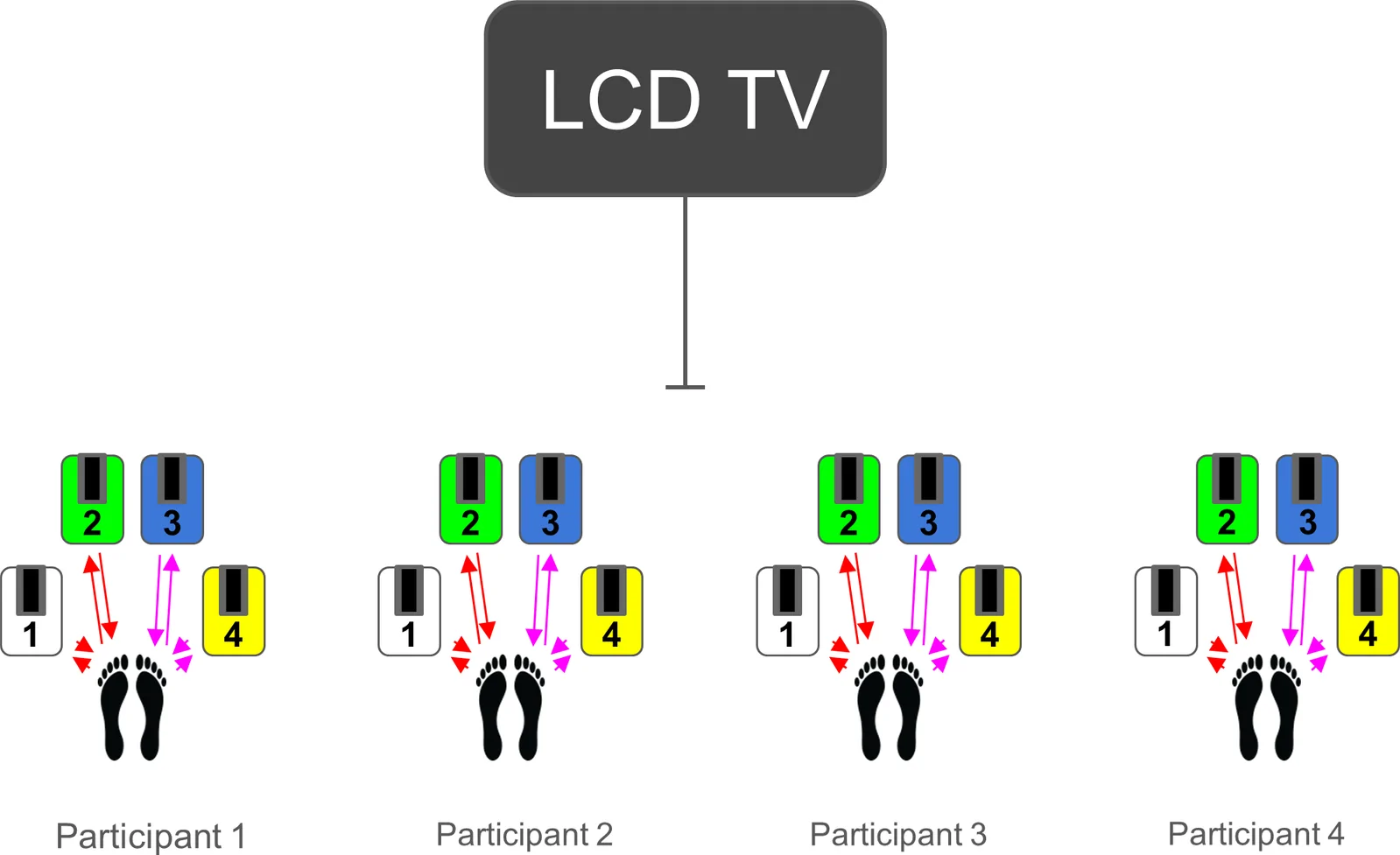
Schematic view of the cognitive training setup for a class of 4 participants from the Activ4Brain group sessions.

For the cognitive training, each participant is assigned a set of 4 pedals connected to a computer that records the pedal presses and execution time (Figure 2). The pedals are USB foot switches for PC computers (FS2017 Pcsensor USB Foot Pedal Control Switch Keyboard Adapter). Each pedal is assigned a number (1-4) and a color (white, green, blue, or yellow). These are fixed throughout the 24 program sessions (Figure 2). The participants stand side-by-side at the front of the room facing an LCD TV screen where task instructions and visual stimuli are displayed in adequate size. Participants are instructed to respond by pressing the pedals according to each task instruction. Detailed descriptions of the cognitive training tasks are included in Multimedia Appendix 1

### Assessment of Performance Changes in the Tasks Used in the Cognitive Training Protocol

To assess if the Activ4Brain intervention improved performance on the trained cognitive tasks, the Activ4Brain group is tested before and after the 3-month program on the first difficulty level of the following tasks: processing speed, inhibitory control, selective attention, working memory, and decision-making. The tests are performed in the laboratory, 1 participant at a time, using the same pedal setup but without music or any other instructed movements besides pedal presses. In these tests, we assess task accuracy, reaction time (time from stimulus onset to movement initiation), and execution time (time from stimulus onset to pedal press). To calculate reaction time, we use triaxial ankle accelerometers (BIOPAC Systems). For logistical reasons, these tests are only applied to the Activ4Brain group and will be used to determine if improvements in the trained tasks mediate the cognitive changes observed in the cognitive assessment protocol.

### Physical Evaluation

Physical evaluation consists of body composition, physical function, and peak oxygen consumption assessment. Tests are performed in the morning between 9 AM and noon, and participants are asked not to participate in vigorous physical activity 24 hours before the tests.

Stature is measured with a stadiometer (Seca Bodymeter, Model 208, Germany) with a precision of 0.1 cm. Body composition (total body mass, fat mass, fat mass percentage, fat-free mass, and phase angle) is measured by bioimpedance (InBody 770; Biospace Co).

To measure the risk of falls and eligibility to perform our exercise programs, TUG, Chair Stand, and Hand Grip Strength tests are applied. TUG and Chair Stand tests are applied according to the Centers for Disease Control and Prevention STEADI guidelines [25,31]. Handgrip strength is measured with a hydraulic hand dynamometer (Lafayette, Model 5030 L1, USA). The strength of the dominant hand is tested in a standing position with straight arms. The test is repeated 3 times, and the best value of the 3 attempts is recorded [32].

Peak oxygen uptake capacity (VO_2_ peak) is measured with a submaximal incremental cycle ergometry test (Lode Excalibur, Netherlands). Following a warm-up period, participants start the testing phase with an initial load of 50 watts for women and 75 watts for men [33]. The load is increased by 25 watts at 3-minute intervals. The test is continued at 60 to 70 rpm and is completed once participants achieve 85% of their predicted maximal heart rate, as determined by the Tanaka formula [34].

### Blood Sample Collection and Analysis

To examine the effect of the intervention on inflammation and neuroprotection blood markers, blood samples are collected from venous blood (15 mL) in a fasting state in the morning by a registered nurse. A complete blood count is performed using a hematology analyzer (DXH500; Beckman Coulter, USA). Aliquots of plasma and serum samples are stored at −80 °C until further use.

Plasma samples will be quantified by enzyme-linked immunosorbent assay for interleukin (IL)-6, IL-1β, tumor necrosis factor-α, interferon-γ, IL-17, IL-1Ra, IL-10, irisin, and brain-derived neurotrophic factor (Thermo Fisher, UK), while serum samples will be analyzed for insulin growth factor 1, vascular endothelial growth factor, and glial cell line-derived neurotrophic factor (ElabScience, USA) according to the manufacturer’s instructions.

### Cognitive Assessment

General cognitive function is assessed using standardized neuropsychological tests (Table 3). Participants are screened for the presence of objective cognitive impairment using the Addenbrooke’s Cognitive Examination-Revised, which also incorporates the Mini-Mental State Examination, providing a double score [35], and for the presence of psychopathology using the Geriatric Depression Scale-30 [36,37]. Participants with objective cognitive deficits (according to the available norms) are excluded.

A comprehensive neuropsychological assessment protocol for detailed neurocognitive performance is performed for eligible participants with 4 tests from the Cambridge Neuropsychological Testing Automated Battery Connect Research system: Motor Screening Task, Rapid Visual Information Processing, Multitasking test, and Spatial Working Memory, and the Auditory Verbal Learning Test [38]. This enabled an in-depth characterization of cognitive function, including measures of attention, visual perception, processing speed, verbal learning, memory, and executive functions, particularly working memory and inhibitory control. Lifestyle habits are screened with the Lifestyle Assessment Toolkit [39]. Physical activity habits are assessed with the International Physical Activity Questionnaire-Short Form [40].

The dementia risk score Lifestyle for Brain Health [41] is applied only at baseline to examine the presence of modifiable risk factors for dementia and, together with the measures International Physical Activity Questionnaire-Short Form, Geriatric Depression Scale, and the Lifestyle Assessment Toolkit, is used to examine potential moderators of program efficacy.

**Table:** **Table 3**. List of neuropsychological assessment measures used.

Number	Neuropsychological assessment protocol	Baseline	Postintervention
1	Informed consent and semistructured interview	✓	
2	Hand and eye laterality tests	✓	
3	Addenbrooke’s Cognitive Examination-Revised test	✓	✓
4	Geriatric Depression Scale	✓	✓
5	CANTAB^a^—Motor Screening Task	✓	✓
6	CANTAB—Rapid Visual Information Processing	✓	✓
7	CANTAB—Multitasking Test	✓	✓
8	CANTAB—Spatial Working Memory	✓	✓
9	Auditory Verbal Learning Test	✓	✓
10	Lifestyle for Brain Health (dementia risk score)	✓	
11	Reduced version of the Healthy Lifestyles Assessment Kit	✓	✓
12	International Physical Activity Questionnaire Portuguese Version (short)	✓	✓
13	Auditory Verbal Learning Test—long-term recall	✓	✓

a CANTAB: Cambridge Neuropsychological Testing Automated Battery.

### Assessment of Brain and Autonomic Function During Rest and Cognitive Task Performance

To examine the effect of the intervention on brain and autonomic function, we record the EEG, the pupilogram, and the ECG while participants are resting and while they are engaged in a value-based decision-making task (reinforcement learning task, described below). For the resting-state recording, participants sit in front of a computer screen and fixate on a target displayed at the center of the screen for 4 minutes.

#### Reinforcement Learning Task

**Figure 3.**
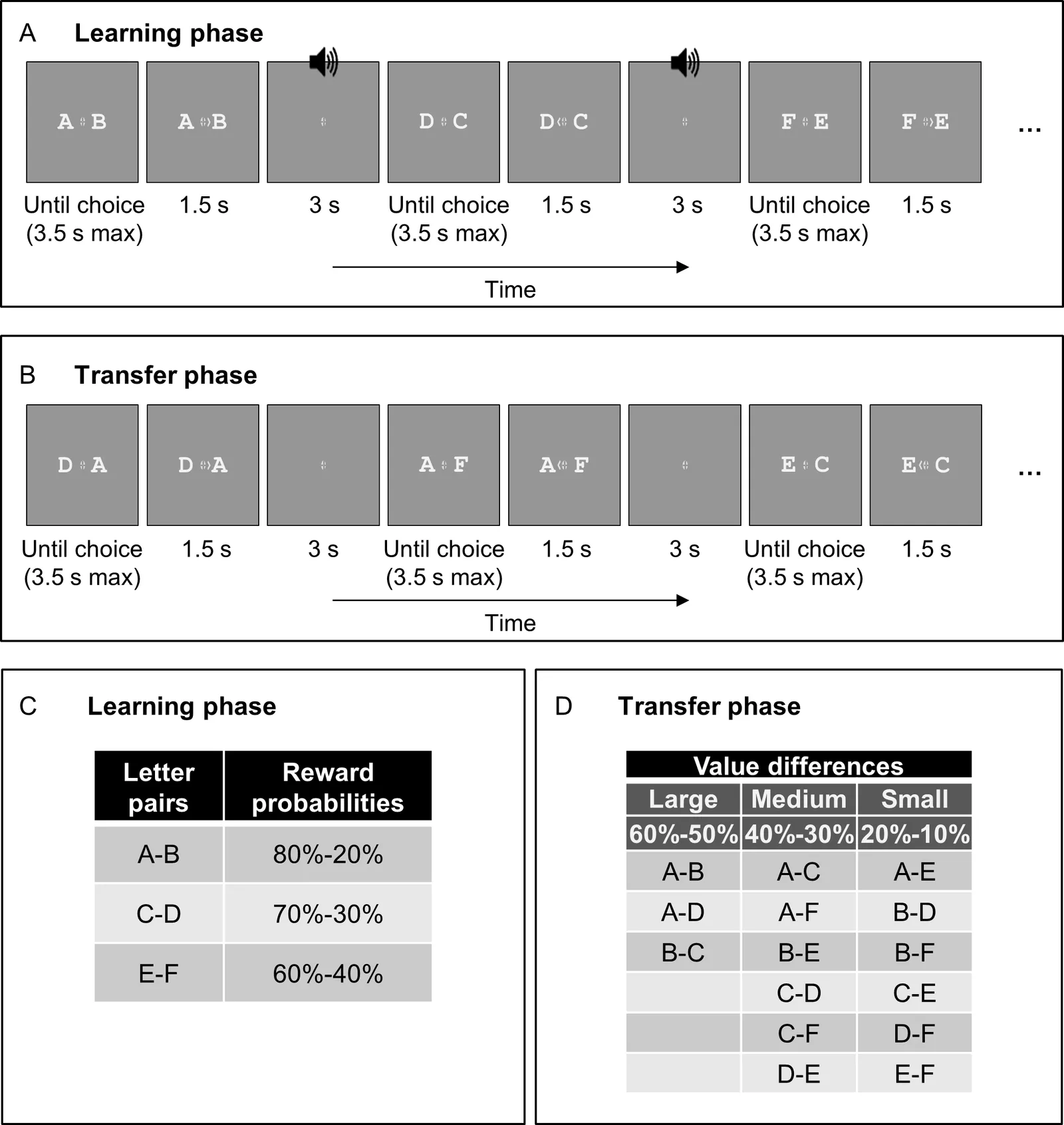
Description of the reinforcement learning task used in the evaluation protocol. (A) During the learning phase, 1 of 3 letter pairs is presented in each trial. Participants choose one of the letters through key press. Each choice is followed by feedback indicating +1 point reward after a rewarded choice or no points for a nonrewarded choice. (B) The transfer phase evaluates how much was learned during the learning phase. All letters are randomly paired with one another, and participants are instructed to select the letter with higher value based on prior learning. Feedback is not presented in the transfer phase. (C) Reward probabilities of the 3 letter pairs used. (D) Value differences of the letter pairs used in the transfer phase.

A probabilistic reinforcement learning task adapted from Van Slooten et al [22] was designed using the Psychophysics Toolbox Version 3 [29,30] for MATLAB. The task consists of 2 main phases: learning and transfer (Figure 3). In the learning phase, 3 pairs of letters from the Latin alphabet (represented here as AB, CD, and EF) are associated with different reward probabilities (80%‐20%, 70%‐30%, and 60%‐40%). At the beginning of each trial, 2 letters appear at the horizontal meridian, left and right from the central fixation target, at 1.15° visual angle from the center of the screen. Participants choose 1 of the letters using the computer keyboard. The choice is highlighted by a small dark gray arrow pointing in the direction of the chosen option (Figure 3). After 1.5 seconds, the letters disappear, and auditory feedback is presented, indicating if the trial was rewarded or not. Auditory feedback consists of the spoken words in Portuguese: “one point” for rewarded trials, “zero points” for nonrewarded trials, or “no response” if the participant did not respond within the time window of 3.5 seconds. The intertrial interval is 3 seconds (from feedback onset to the start of the next trial). In the transfer phase, all 6 letters used are mixed to form 15 letter pairs (AB, AC, AD, AE, AF, BC, BD, BE, BF, CD, CE, CF, DE, DF, EF). Participants are asked to choose the letter with the highest reward based on previous learning. No feedback is provided during the transfer phase. Trials of the transfer phase follow the same trial structure as trials in the learning phase, except that no feedback is provided. Each session starts with a training block designed to explain the aims of the task, ensuring the participants understand the task rules and aims clearly. The learning phase consists of 4 runs and 90 trials per run, 360 trials in total. After each run, the number of points obtained is displayed. Directly after the learning phase, participants enter the transfer phase. The transfer phase consists of 3 runs and 60 trials per run, 180 trials in total. In the transfer phase, overall choice accuracy (percentage of times participants choose the letter associated with higher reward probability) is displayed only at the end of the recording session.

Participants are seated in a dimly lit room with their head positioned on a chin rest, 80 cm away from the computer screen. The letters (width × height=0.51° × 0.76° visual angle) are presented in a dark gray color on a lighter gray background (Figure 3). To minimize eye movements, participants are instructed to fixate on a fixation target displayed at the center of the screen throughout the whole task [42]. The luminance of the background, letters, and the fixation target are 47, 31, and 39 cd/m², respectively. Six different stimuli (6 letters) are randomly assigned for each participant and for each session (baseline and follow-up), corresponding to a total of 12 different letters assigned randomly for each participant.

#### EEG, ECG, and Eye Tracking Data Acquisition

A 64-channel Neuroscan system (Compumedics Europe GmbH) is used to measure EEG and ECG. The scalp electrodes are placed according to the International 10‐20 placement standard, with reference between the electrodes CPz and Cz and ground between FPz and Fz. The acquisition sampling rate is 500 Hz. Vertical and horizontal electrooculograms are recorded to monitor eye movements and blinks. Bipolar ECG electrodes are placed on the right shoulder and at the bottom of the sternum.

The EyeLink 1000 Plus desktop mount (SR Research) eye-tracking system is used to record the horizontal and vertical gaze positions and the pupilogram, with a sampling rate of 500 Hz. Eye position calibration and validation are run before each run. The participant’s head position is stabilized by using a chin rest to help reduce artifacts in the gaze signal that can be caused by head movements.

Trigger pulses are generated at the onset of each stimulus (letter pairs and auditory feedback) and at every button press.

### Adherence Rate and Adverse Events

Participants’ attendance is recorded for each exercise session. If a participant does not attend 2 consecutive exercise sessions, 1 researcher contacts the participant and encourages them to continue attending the classes. An adherence rate of 60% is necessary for inclusion in the analyses [43].

To ensure maximum safety, the following measures are taken: heart rate monitoring, performing exercise on a soft ground, and exclusion of participants with any health issue not compatible with moderate AE. Any adverse events that occur during the exercise program and study procedures are recorded by the researchers.

### Exit Interviews

At follow-up, the AE and Activ4Brain groups respond to custom-made satisfaction questionnaires and system usability surveys [44-46]. These questionnaires and surveys include questions regarding suggestions for program improvement and scales regarding their perception of the impact of the program on their well-being and their satisfaction with the program.

### Study Outcomes

#### Assessment Procedures

Study outcomes are assessed at baseline and after the end of the intervention period (3-mo follow-up). Each assessment time point comprises 4 facility-based visits: (1) cognitive assessment (2 h), (2) EEG, pupil, and ECG recording session (2 h), (3) physical evaluation session (30 min), and (4) blood collection. Participants from the Activ4Brain group attend an additional visit to be assessed on the cognitive training tasks used in the program (30 min). The testing sessions end prematurely if the participants show signs of fatigue. Participants are informed of the preparation requirements, which will be checked prior to the assessments.

The study is designed to be powered for the primary outcome only (cognitive function). All secondary and other outcomes—brain and autonomic function (EEG, pupil, ECG), inflammatory and neuroprotective markers, physical fitness and body composition, feasibility and usability, and analyses of the associations between changes in these markers and changes in cognitive function—are exploratory. They are not individually powered and are intended to generate new hypotheses and inform the design of future, adequately powered studies.

#### Primary Outcomes

The primary aim of this study is to assess whether the Activ4Brain program leads to improved cognitive function in cognitively healthy older people. We will focus on measures from the cognitive assessment protocol associated with 5 cognitive domains: executive function, working memory, episodic memory, sustained attention, and processing speed. These are our primary outcomes.

#### Secondary Outcomes

Secondary outcomes include:

1. Evaluation of the impact of the Activ4Brain program on brain and autonomic function and physical health markers, including molecular changes associated with inflammation and neuroprotection, and investigation of potential moderators of the program’s efficacy
2. Evaluation of participants’ adherence and retention through attendance rates across all intervention sessions and participation trends over time
3. Evaluation of the feasibility of the Activ4Brain program, including its acceptability, accessibility, and usability

#### Other Outcomes

Other outcomes include evaluation of changes in:

1. Cardiovascular and physical functional fitness and body composition, which are important for preventing chronic diseases and age-related muscle loss and are associated with cognition
2. Physical activity participation, which is especially important in older people who have a sedentary lifestyle;
3. Psychosocial measures and quality of life to investigate if our group-based intervention positively influences the mental health and emotional well-being of the participants, for example, by reducing loneliness and isolation.

### Statistical Analysis Plan

The study of the effect of the Activ4Brain program on the primary outcome (cognitive function) is confirmatory because we hypothesize, according to previous evidence, that the integrated cognitive-aerobic training program results in larger improvements than AE alone or no intervention (passive control) [47]. All other analyses are exploratory.

Continuous cognitive outcomes will be summarized descriptively with mean (SD) for each group and time. These variables will be analyzed with repeated-measures ANOVA with 1 between-subjects factor (group) and 1 within-subjects factor (time=preintervention vs postintervention). The Activ4Brain intervention will be considered effective in improving cognitive function if the group × time interaction is significant at a level of .05, if the post hoc tests (with CI adjustment using Bonferroni) show a significant effect of time for the Activ4Brain group, and the improvement is higher for this group than for the other control groups. If the data do not meet assumptions of sphericity, the Greenhouse-Geisser correction will be used. Description of the effect sizes by partial η^2^ will follow the following cutoff points: less than 0.06 will be considered a small effect; between 0.06 and 0.14 will be considered a medium effect; and higher than 0.14 will be considered a large effect [48].

Missing data will be assessed prior to analysis. Analyses will be conducted using the available data (complete case analysis), and participants with missing data for a specific outcome variable will be excluded only from the relevant analysis.

### Data Management and Monitoring Plan

A unique code will be attributed to each participant, and the data will be registered in databases separate from any identifiable information. For each stage of data collection, the validity and integrity of the collected data will be checked by at least 2 researchers. The researchers will validate data ranges and cross-check the entries in the databases.

Due to the trial’s short duration and minimal risks, there will be no requirement for a Data Monitoring Committee or interim analyses.

### Data Sharing

Twelve months after each publication of trial results, the deidentified individual participant data that underlie the published results will be made available indefinitely in a public repository (eg, Open Science Framework, OpenNeuro) to anyone who wishes to use the data for any purpose.

## Results

Data collection started in January 2024 and finished in April 2025. Seventy-three individuals (40 women and 33 men) were enrolled in the study and assigned to the 3 groups. The average age was 66 (SD 6) years. Data analyses are ongoing, with the publication of the results estimated for 2026 and 2027.

## Discussion

Enhancing the levels of physical and cognitive function of older people can have a significant effect on their quality of life. The Activ4Brain program aims to achieve this as a group-based AE program that incorporates targeted cognitive training and is designed to be fun and engaging and easily applicable in the community or in care institutions.

This study will allow us to determine if this program has a positive effect on the cognitive function of older people and compare its effectiveness with that of AE alone. Additionally, we will investigate the neurobiological mechanisms underlying the program’s impact on cognition by studying the associations between brain, autonomic, and physical function and inflammation and neuroprotective markers. Understanding these associations is essential to understand the mechanisms by which exercise and cognitive training may help preserve cognition and will contribute to the refinement and targeting of existing interventions [2]. Specifically, pupil-linked arousal responses and EEG measures of brain function will be used to elucidate the neurobiological mechanisms underlying cognitive changes. Engagement of pupil-linked arousal during exercise has been shown to correlate with improvements in cognitive task performance [49,50]. In fact, a single bout of exercise can change the engagement of the pupil-linked arousal system during cognitive tasks, leading to performance improvements [51]. Thus, engagement in regular exercise combined with cognitive training might change the way the pupil-linked arousal system is activated during cognitive task performance, and this might positively impact brain function and behavior. The data acquired within the scope of this study protocol will allow us to evaluate these hypotheses.

Neuroprotective and inflammatory biomarkers will be studied to investigate the neurobiological mechanisms of the effect of exercise and cognitive training on cognition, thus contributing to the understanding of muscle-immune-brain interactions. Aging-related increases in low-grade chronic inflammation, associated with immunosenescence, may lead to neurodegeneration and cognitive decline [52-55]. Circulating proinflammatory cytokines such as IL-1β, tumor necrosis factor-α, and IL-6 can activate microglia and astrocyte cells and elevate production of proinflammatory cytokines in the brain, which causes neuronal death and cognitive decline [56]. Exercise has anti-inflammatory effects [57-60], induces a decrease in proinflammatory monocytes, and causes an acute increase in muscle-secreted IL-6 that elevates the secretion of anti-inflammatory cytokines like IL-10 and IL-1RA [58]. Moreover, cognitive stimulation also decreases inflammatory cytokine levels in older populations [61]. Thus, the combination of exercise and cognitive stimulation may have synergistic benefits on the inflammatory profile.

One of the strengths of this intervention is that, unlike earlier exergaming and dual-task programs that rely on limited cognitive tasks or fixed difficulty, Activ4Brain targets 5 aging-sensitive cognitive domains, adapts task difficulty weekly to performance, and delivers cognitive training during exercise-induced arousal, in a socially engaging group format intended to support long-term adherence.

The retrospective nature of the publication of this protocol has the objective of providing a detailed description of the methods, ensuring that the information is available for trial replication. However, due to the inherent limitation associated with the fact that the publication of the protocol occurred after the end of data collection and the analysis plans presented were not specified before the data were acquired, the results from the study will be used mainly to generate new hypotheses for future clinical trials. Nevertheless, we expect that integrated cognitive and AE will have a stronger effect on cognition than AE alone [47], and we will treat this analysis as confirmatory. However, it is important to note that we are studying the effects of a novel intervention designed specifically for this study, and we do not have strong convictions regarding which specific cognitive domains will be most affected and which cognitive tests will best capture these effects, particularly when addressing cognitive decline in healthy older people. In fact, a large percentage of studies investigating the impact of this type of intervention on cognition use measures of global cognition such as the Mini-Mental State Examination or the Montreal Cognitive Assessment. These tests, while adequate to help diagnose mild cognitive impairment and dementia, suffer from ceiling effects in the cognitively healthy population [62]. To avoid this problem, this protocol includes measures of cognition more sensitive to subtle changes in cognitively healthy older adults.

Notably, it is possible that this type of intervention might be particularly effective in people with lower physical function and/or lower cognitive function who present a higher potential for improvement. In fact, there is evidence that age-related cognitive decline shows high variability, with some individuals aging better than others [63]. Future studies should explore the impact of the intervention focusing on populations with sedentary lifestyles, lower education levels, or poorer cognitive function.

Finally, the study was designed to include many outcomes aimed at studying not only the impact of the program but also the neurobiological mechanisms underlying its effects. However, the sample size was calculated to test only the primary outcome (cognitive function). This limitation results in all the other analyses being exploratory, so these results will not confirm the additional benefits of the Activ4Brain program but will rather contribute to the formulation of new hypotheses for future studies.

In conclusion, the Activ4Brain program offers a promising intervention that takes advantage of the known neurobiological impact of AE and cognitive training to potentiate their impact on cognition. The gamification of the computerized cognitive training tasks specifically designed to target relevant cognitive domains and the organization of the training in group classes has the potential to increase user motivation and long-term adherence, potentiating the program’s impact. The findings of this trial will contribute to our understanding of the best interventions to improve cognitive function in old age and will contribute toward our understanding of the neurobiological mechanisms underlying the impact of exercise and cognitive training on cognitive, brain, mental, and physical health.

## Supplementary material

10.2196/92631 Multimedia Appendix 1 Cognitive tasks included in the Activ4Brain program.

10.2196/92631 Checklist 1 SPIRIT 2025 checklist.

## Abbreviations

AE: aerobic exercise
CONSORT: Consolidated Standards of Reporting Trials
ECG: electrocardiogram
EEG: electroencephalogram
IL: interleukin
SPIRIT: Standard Protocol Items: Recommendations for Interventional Trials
TUG: Timed Up and Go
