# Supplementary material for: The Effect of Integrated Aerobic and Cognitive Training (Activ4Brain) on Cognition and Neurophysiology in Older Adults: Protocol for a Controlled Trial
Source: JMIR Res Protoc. 2026 Sep 17;15:e92631. doi: 10.2196/92631 (PMC13585018; doi:10.2196/92631)
Supplement: Supplementary file 1 [file resprot-v15-e92631-s001.pdf]

The Effect of Integrated Aerobic and Cognitive Training (Activ4Brain) on Cognition and Neurophysiology in Older Adults: Protocol for a Controlled Trial

Hicran Besikci, Kaan Akalp, Luís Rama, Beatriz Branquinho Gomes, José Pedro Leitão Ferreira, Susana Mouga, Inês Bernardino, Miguel Castelo-Branco, Ana Rita Sousa e Silva, Ana Maria Teixeira, Maria José Ribeiro

SUPPLEMENTAL MATERIALS

Cognitive Training Tasks Included in the Activ4Brain Program

The Activ4Brain program includes cognitive training tasks targeting five cognitive domains: processing speed, inhibitory control, selective attention, working memory and decision-making, designed using the Psychophysics Toolbox Version 3[1,2] in MATLAB (MathWorks Inc.). For task performance, each participant is assigned a set of four pedals connected to a computer that records the pedal presses and execution time (Suppl. Fig. 1). Each pedal is assigned a number (1 to 4) and a color (white, green, blue or yellow), and these are fixed throughout the 24 program sessions. The training is performed in group classes of 4 participants that stand side-by-side at the front of the room facing an LCD TV screen where task instructions and visual stimuli are displayed in adequate size clearly visible by all participants. Participants are instructed to respond by pressing the pedals according to the task instructions.

Every week, the cognitive training tasks' difficulty level is increased if the group class average accuracy for the previous week exceeds 80%. The following sub-sections provide detailed descriptions of the cognitive training tasks and their difficulty levels.

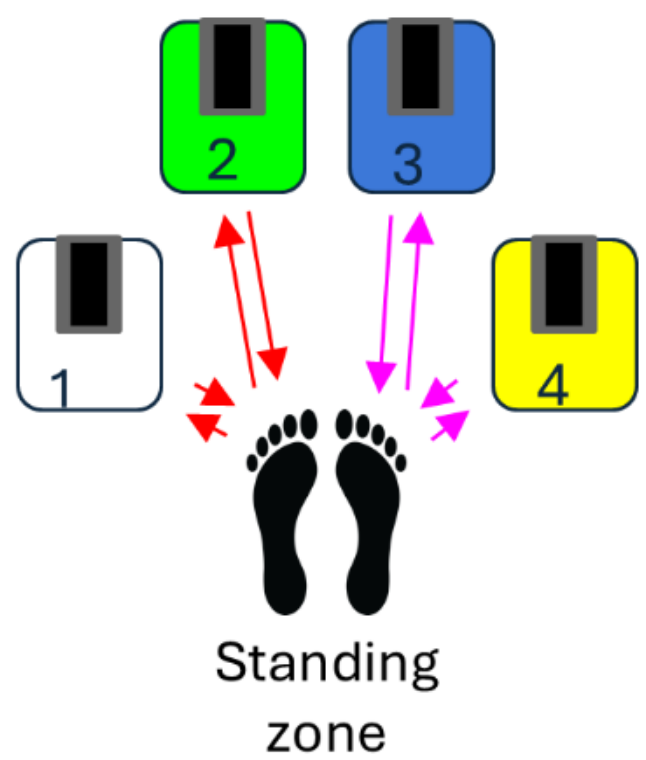

Supplementary Figure 1. Schematic view of the response pedals set-up for the cognitive training component of the Activ4Brain program.

1. Processing Speed Training Task

In the Processing Speed Training Task, participants are shown a random sequence of colors (white, green, blue or yellow) and/or numbers (1 to 4) and are instructed to respond by pressing the corresponding pedals as accurately and as fast as possible. The task lasts five minutes and is divided into smaller blocks of 30 s. At the beginning of each block, a written instruction indicates the type of stimuli that will be presented in the following block of trials (Suppl. Fig. 2). Stimuli are presented sequentially with a grey screen in between. Participants' responses are recorded until the next stimulus appears on the screen. The task has three difficulty levels. After the third level, this task is substituted by the decision-making training task.

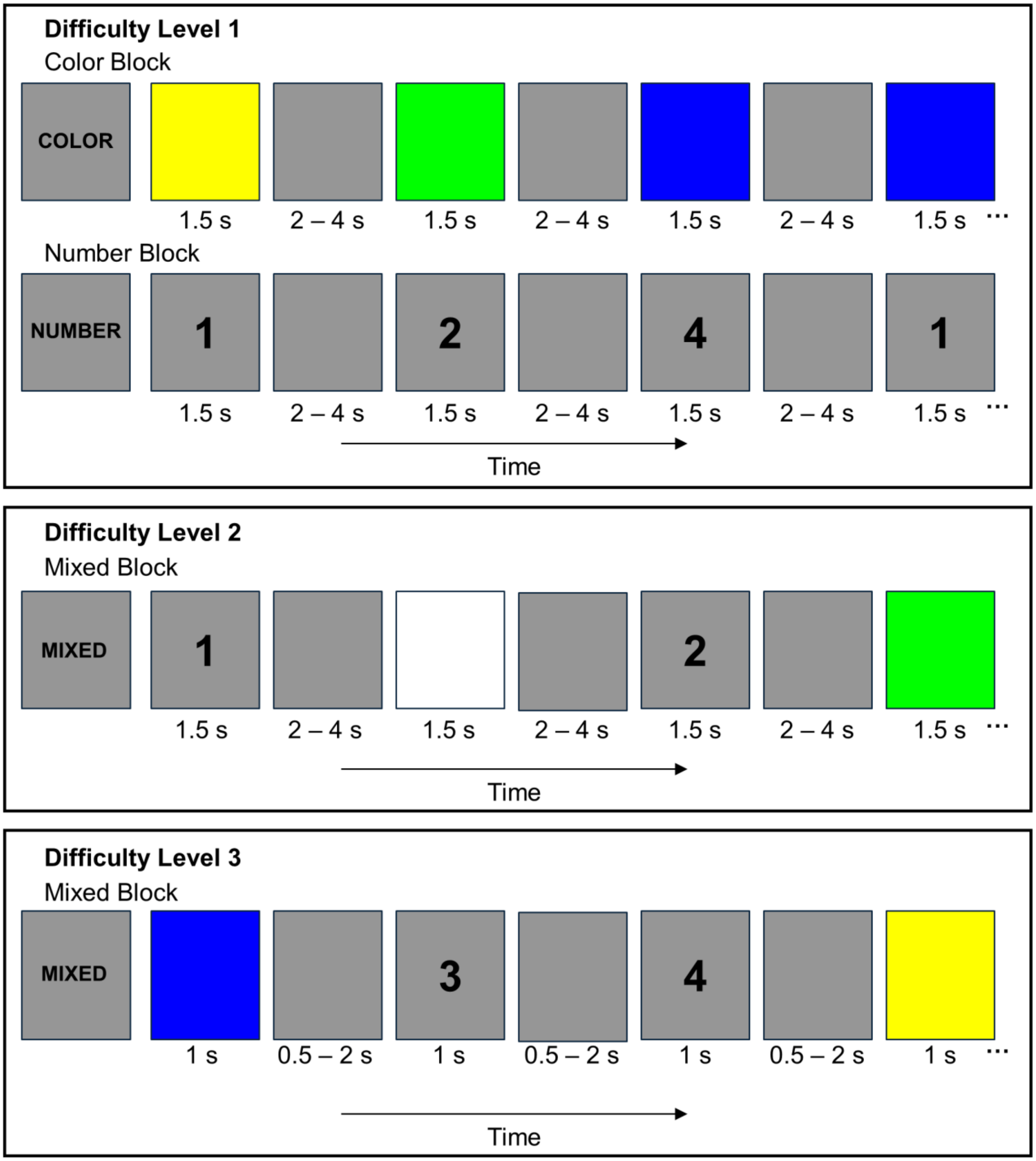

Supplementary Figure 2. Schematic representation of the Processing Speed Training Task (not to scale). The upper panel represents the sequences used in difficulty level 1 where colors and numbers are displayed in separate blocks. The middle and the lower panels represent the sequences used in difficulty levels 2 and 3, respectively. In these levels, the sequences contain both color and number stimuli.

The following 3 difficulty levels are implemented for the Processing Speed Training Task.

Level 1 - Each 30s-block includes a sequence of numbers or colors. Number and color blocks are presented in an alternating manner. Stimulus duration is 1.5 s and the interstimulus interval (ISI) is a random interval between 2 and 4 s.

Level 2 – Mixed blocks. Each block consists of a sequence containing an equal number of color and number items presented in random order. Stimulus duration is 1.5 s and the ISI is a random interval between 2 and 4 s.

Level 3 - Mixed blocks and faster stimulus presentation. Each 30s-block consists of a sequence containing an equal number of color and number items presented in random order. The stimulus duration is 1 s and the ISI is a random interval between 0.5 and 2 s.

2. Inhibitory Control Training Tasks

The program Activ4Brain included three cognitive tasks designed to train inhibitory control: two go/no-go tasks and one stop signal task. These are described in detail in the following sub-sections.

2.1. Colors and Numbers Go/No-go Task

The Colors and Numbers Go/No-go Task is based on the design of a go/no-go task, lasts 5 min and is divided into 40 s blocks. At the beginning of each block, an instruction is presented for 2 s identifying the go and the no-go stimuli (Suppl. Fig. 3). After this screen, a sequence of colors (white, green, blue or yellow) or numbers (1 to 4) is presented. Participants are instructed to step on the corresponding pedal when a go stimulus is presented and inhibit the response when a no-go stimulus is presented. Go stimuli are more frequent than no-go stimuli. For each block, the number of no-go stimuli is randomly determined to range between 1 and 4, corresponding to approximately 10 to 40% of the total number of stimuli within the block. Each trial consists of the presentation of a stimulus followed by a grey screen. Participants' responses are recorded during the whole trial duration.

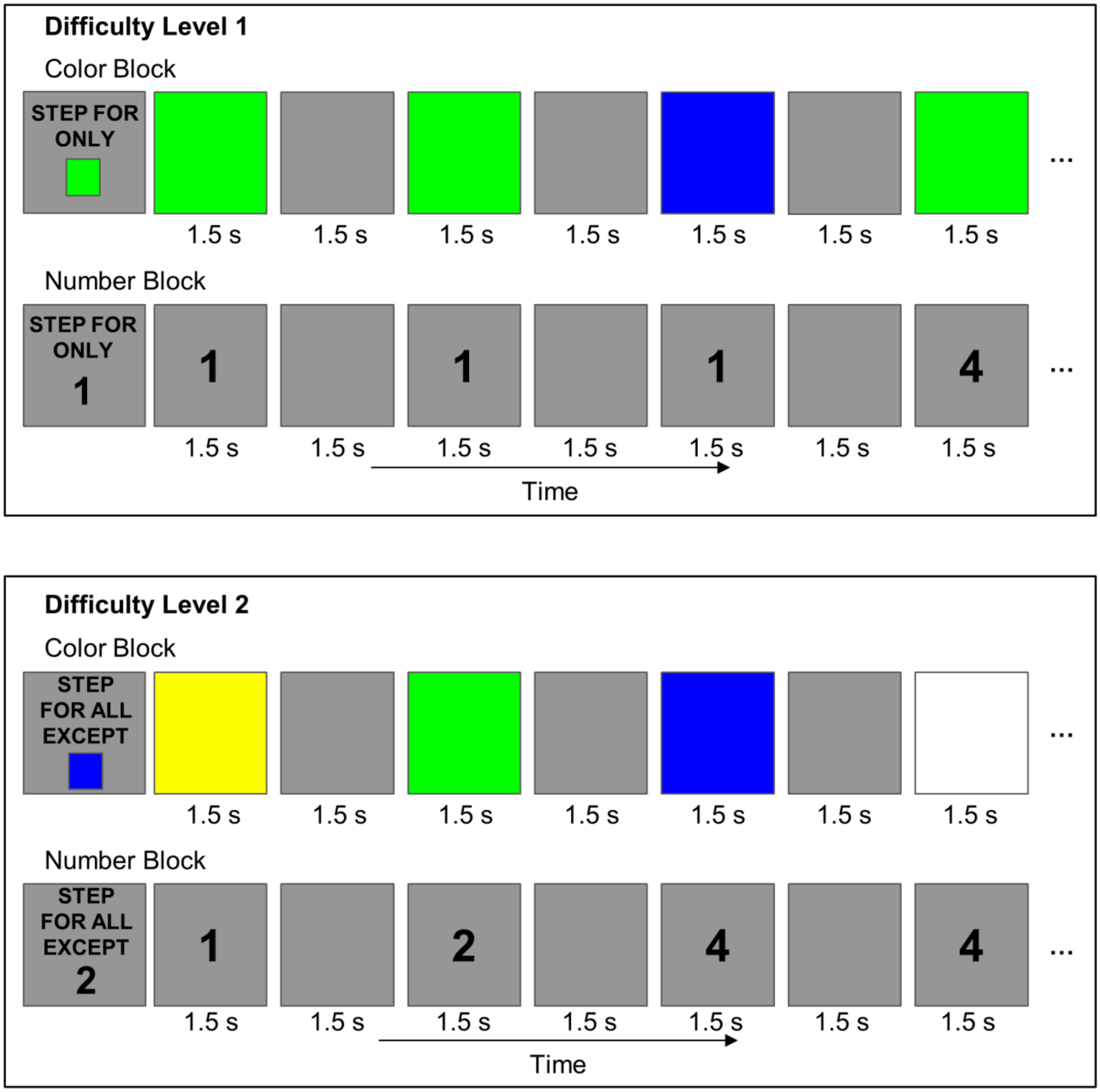

Supplementary Figure 3 – Schematic representation of the Colors and Numbers Go/No-go Task (not to scale). Sequences of color or number stimuli are displayed on the screen and participants are instructed to respond to the go stimuli by pressing the corresponding pedal and withhold their responses when the no-go stimuli are displayed. In difficulty level 1 (top panel), at the beginning of each block, a color or a number is shown as the go stimulus. The other three stimuli are implicitly assigned as no-go stimuli. In the difficulty level 2 (bottom panel), at the beginning of each block, a color or a number is shown as the no-go stimulus. The other three stimuli are implicitly assigned as go stimuli.

The following 4 difficulty levels are implemented for the Colors and Numbers Go/No-go Task.

Level 1 – In this level, only one color or number is the go stimulus. At the beginning of each block, an instruction panel reveals which color or number is the go stimulus. The other three colors/numbers are implicitly assigned as the no-go stimuli. Each block contains 10 stimuli. The duration of the stimuli is 1.5 s and the ISI is 1.5 s.

Level 2 - In this level, instead of having several no-go stimuli and one go stimulus, we have one no-go stimulus and several go stimuli. At the beginning of each block, an instructions panel reveals which color or number is the no-go stimulus. The other three colors/numbers are implicitly assigned as go stimuli. Each sequence contains 11 stimuli. The duration of the stimuli is 1.5 s and the ISI is 1.5 s.

Level 3 – In this level, the task speed is increased. At the beginning of each block, an instructions panel reveals which color or number is the no-go stimulus. The other three colors/numbers are implicitly assigned as the go stimuli. Each sequence contains 10 stimuli. The duration of the stimuli and the ISI are decreased to 1 s.

Level 4 - In this level, the task speed is increased again. At the beginning of each block, an instructions panel reveals which color or number is the no-go stimulus. The other three colors/numbers are implicitly assigned as the go stimuli. Each sequence contained 10 stimuli. The duration of the stimuli and the ISI are decreased to 0.75 s.

2.2. Stop Signal Task

This task is an adaptation of the stop signal task. The task duration is 5 min, divided into 8 blocks of approximately 40s each. In each block, a sequence of colors (white, green, blue or yellow) and/or numbers (1 to 4) is presented, and the participants are instructed to press the corresponding pedal except in the trials where a stop sign (a stop hand image) is presented over the stimulus (Suppl Fig. 4). The stop sign is always presented after stimulus onset with a variable delay. Difficulty is manipulated by reducing the number of stimuli with stop signs and increasing the time between stimulus onset and the stop signal increasing the need to engage inhibitory control.

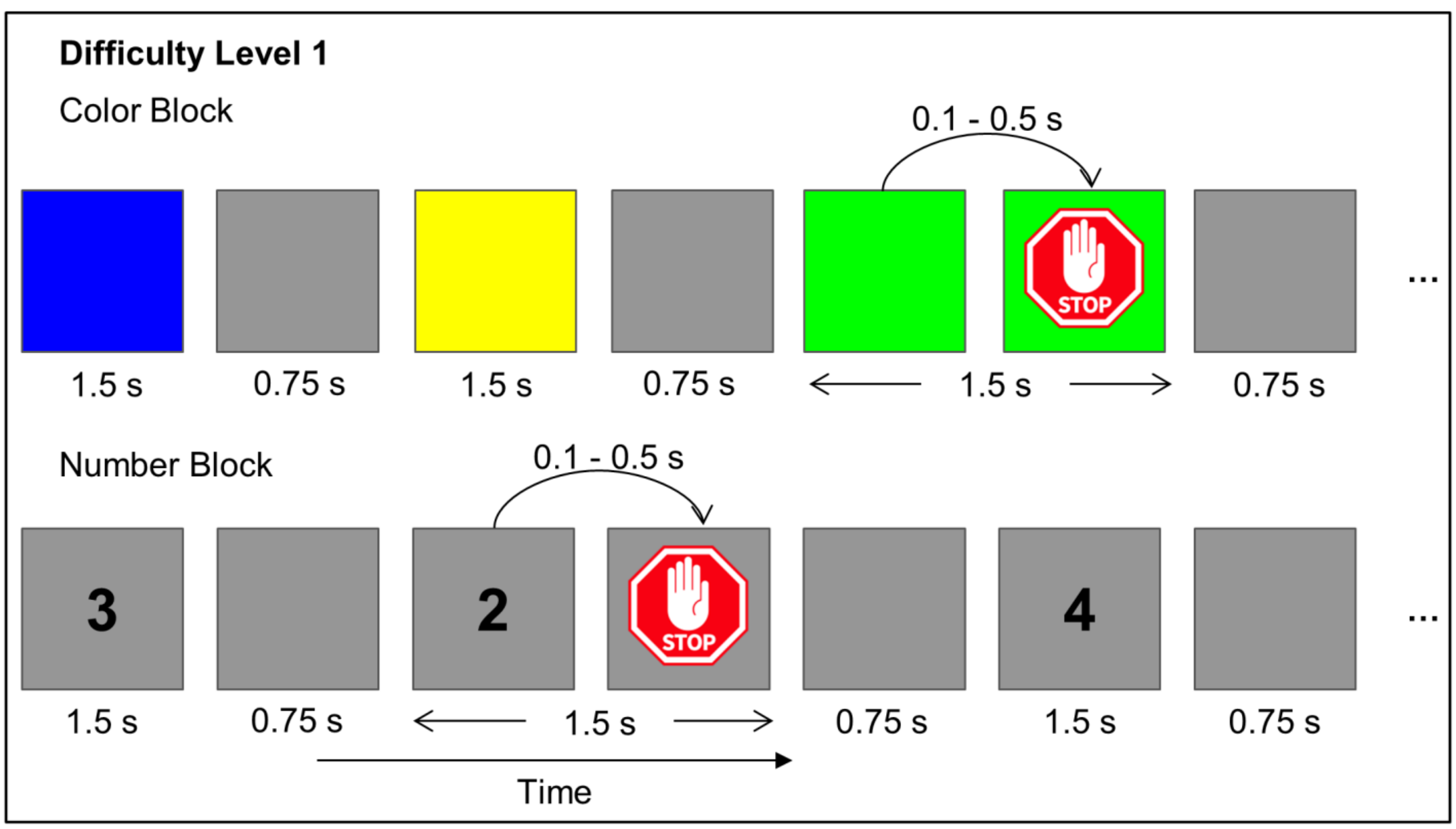

Supplementary Figure 4 – Schematic representation of the Stop Signal Task. Each block consists of a sequence of colors or numbers (not to scale). The stop sign appears on the screen as an inhibition stimulus at a random time window after the color or number stimulus onset. Participants are instructed to step on the corresponding pedal for all stimuli except if the stop sign is presented. In those trials, they should withhold their response.

The following 4 difficulty levels are implemented for the Stop Signal Task.

Level1 – Each block contains 20 stimuli in total. Two blocks contain 7 stop signals, two blocks contain 6 stop signals, two blocks contain 5 stop signals and two blocks contain 4 stop signals. The order of the blocks is randomized. The stimuli duration is 1.5 s and the ISI is 0.75 s. The time between stimulus onset and the stop sign is between 0.1 s and 0.5 s, randomly assigned.

Level 2 - In this level, the frequency of stop signals is decreased to render them more unexpected. Each block contains 18 stimuli. Four blocks contain 5 stop signals, two blocks contain 4 stop signals, and two blocks contain 3 stop signals. The order of the blocks is randomized. The stimuli duration is 1.5 s and the ISI is 0.75 s. The time between stimulus onset and the stop sign is between 0.1 and 0.5 s, randomly assigned.

Level 3 – In this level, the delay between stimulus onset and the stop sign is increased. Each block contains 18 stimuli. Four blocks contain 5 stop signals, two blocks contain 4 stop signals, and 2 blocks contain 3 stop signals. The order of the blocks is randomized. The stimuli duration is 1.5 s and the ISI is 0.75 s. The time between stimulus onset and the stop sign is between 0.2 and 0.7 s, randomly assigned.

Level 4 – In this level, task speed is increased. Each block contains 21 stimuli. Three blocks contain 5 stop signals, three blocks contain 4 stop signals, and two blocks contain 3 stop signals. The order of the blocks is randomized. The stimuli duration is 1s and the ISI is 0.75 s. The time between stimulus onset and the stop sign is between 0.2 and 0.7 s, randomly assigned.

2.3. Stars and Bombs Go/No-go Task

The Stars and Bombs Go/No-go Task lasts 5 min and is divided into 8 blocks of 18 trials each. Throughout each block, an image containing 4 black rectangles, matching the position of the participant's pedals, is presented on the screen. In each trial, over this image, one of 2 different types of stimuli is presented on top of one of the represented pedals: stars (frequent go stimuli) or bombs (rare no-go stimuli) (Suppl. Fig. 5). Participants are instructed to catch all the stars by pressing the corresponding pedal before the star disappears and to avoid responding to the bombs. Four blocks contain 5 no-go stimuli, two blocks contain 4 no-go stimuli, and two blocks contain 3 no-go stimuli. The blocks were presented in randomized order.

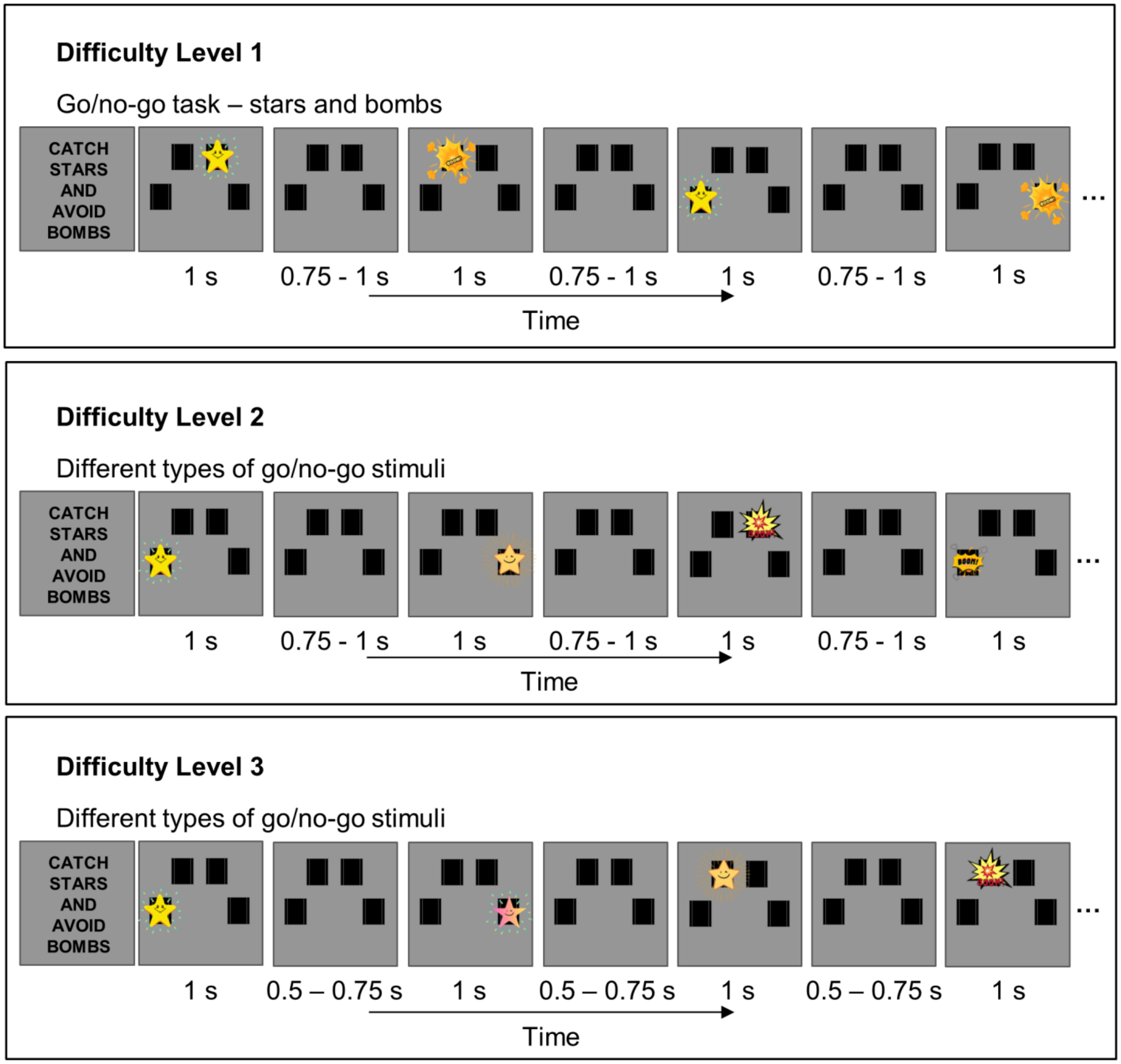

Supplementary Figure 5. Schematic representation of the Stars and Bombs Go/No-go Task (not to scale). Starts (frequent go stimuli) or bombs (rare no-go stimuli) are displayed over an image of black rectangles representing the 4 responding foot pedals. Participants are instructed to press the corresponding pedal when a start is presented and withhold the foot response when a bomb is presented.

The following 3 difficulty levels are implemented for the Stars and Bombs Go/No-go Task.

Level 1 – In this level, only one type of start and one type of bomb are used as the go and the no-go stimulus. The stimulus duration is 1s and the ISI is randomly defined between 0.75 s and 1 s.

Level 2 – In this level, difficulty is increased by using three different types of stars as go stimuli and three different types of bombs as no-go stimuli, thereby requiring higher attention to the stimulus features. The stimulus duration is 1 s and the ISI is randomly defined between 0.75 s and 1 s.

Level 3 - In this level, task speed is increased. Three different types of stars are used as go stimuli and three different types of bombs as no-go stimuli. The stimulus duration is 1s and the ISI is randomly defined between 0.5 s and 0.75 s.

3. Selective Attention Training Tasks

Two tasks are used to train selective attention: a Stroop Task and a Concentration Grid Task described below.

3.1. Stroop Task

This task is an adaptation of the Stroop task, lasts 5 minutes and is divided into blocks of 40s. In this task, stimuli are numbers (1 to 4) printed in white, green, blue or yellow. The number and color can be congruent with the pedal number and color assignment (Suppl. Fig. 1) or incongruent.

At the beginning of each sequence, participants are instructed to focus only on the color or on the number and respond as fast as possible by pressing the corresponding pedal (Suppl. Fig. 6). Participants' responses are recorded until the beginning of the next trial. In half of the blocks, participants are instructed to respond according to the color and in the other half are instructed to respond according to the number.

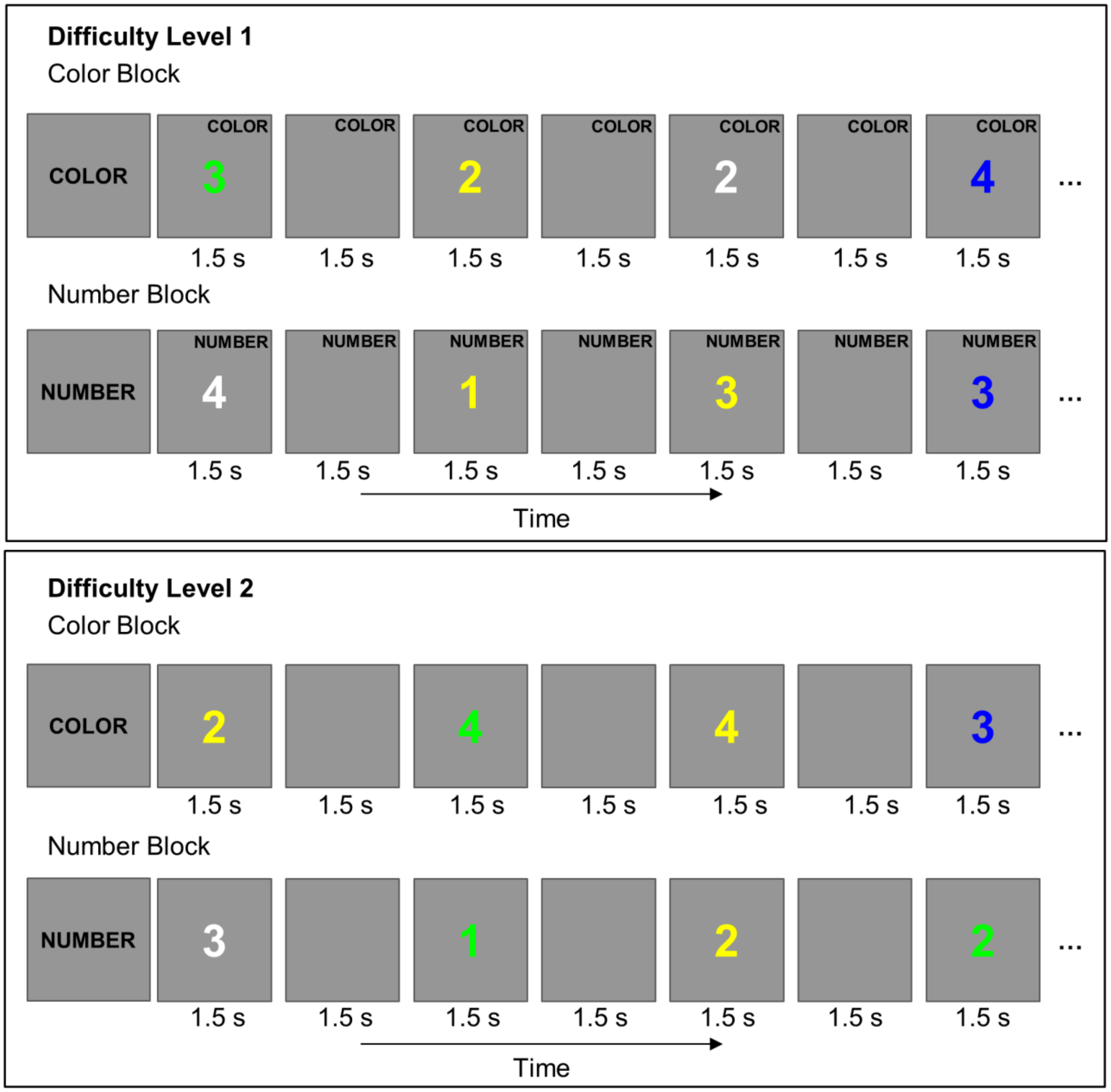

Supplementary Figure 6. Schematic representation of the structure of the Stroop Task (not to scale). In this task, numbers (1 to 4) are displayed in different colors (green, white, yellow or blue). The first panel of each block instructs the participants to focus on either the color or the number displayed and respond by pressing the corresponding pedal as fast as possible. In the difficulty level 1 represented in the upper panel, the stimulus characteristic that they need to pay attention to is indicated as a hint in the right corner of the screen to assist with task performance. In the difficulty level 2, represented in the lower panel, the hint is removed increasing the requirements for working memory.

The following 4 difficulty levels are implemented for the Stroop Task.

Level 1 – This level contains 8 blocks of 10 stimuli each. The stimulus duration is 1.5 s and the ISI is 1.5 s. A hint is presented in the right corner of the screen to remind which stimulus characteristic the participants should pay attention to (color or number).

Level 2 – In this level, the hint is removed, and the participants are required to keep it in memory. This level contains 8 blocks of 10 stimuli each. The stimulus duration is 1.5 s and the ISI is 1.5 s.

Level 3 – In this level, task speed is increased. This level contains 12 blocks of 10 stimuli each. The stimulus duration is decreased to 1 s and the ISI is 1 s.

Level 4 – In this level, the number of stimuli per block is increased requiring additional working memory to maintain task instructions online. This level contains 8 sequences of 15 stimuli each. The stimulus duration is 1 s and the ISI is 1 s.

3.2. Concentration Grid Task

This is a visual search attention task, adapted from a concentration grid task[3,4]. In each trial, a NxM grid of squares is displayed with numbers or colors embedded within individual squares (Suppl. Fig. 7). During each trial, one of the 4 possible colors (white, green, blue or yellow) or numbers (1 to 4) is omitted. The task is divided into blocks of trials where the participants are instructed to find the missing color (color blocks) or the missing number (number blocks) and press the corresponding pedal as fast as possible.

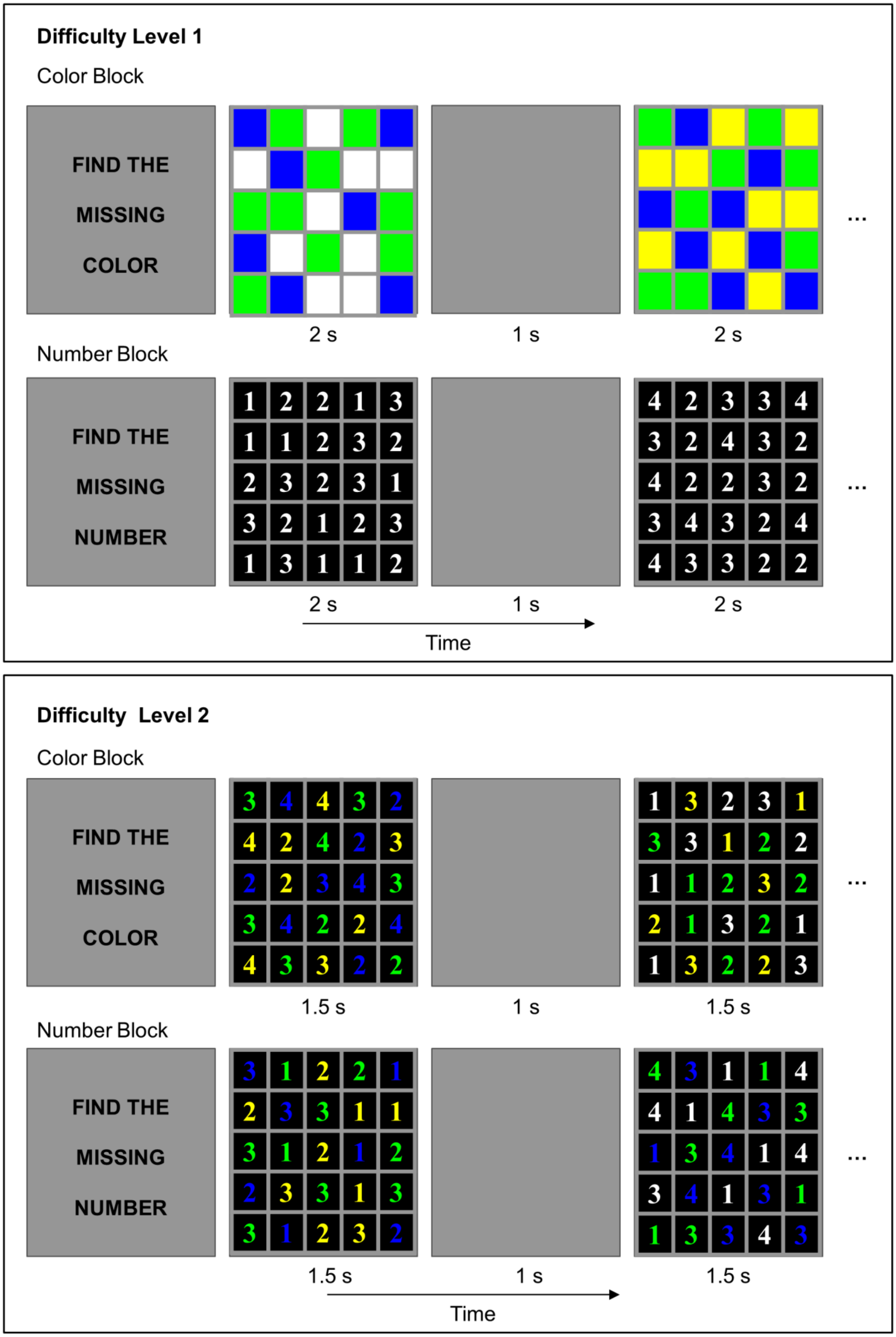

Difficulty Level 1
Color Block
FIND THE MISSING COLOR
2 s
1 s
2 s
...
Number Block
FIND THE MISSING NUMBER
2 s
1 s
2 s
...
Time
Difficulty Level 2
Color Block
FIND THE MISSING COLOR
1.5 s
1 s
1.5 s
...
Number Block
FIND THE MISSING NUMBER
1.5 s
1 s
1.5 s
...
Time

Supplementary Figure 7. Schematic representation of the Concentration Grid Task (not to scale). In each trial, a NxM square grid is displayed with numbers or colors embedded within individual squares. During each trial, one of the four possible colors or numbers is omitted. Participants are instructed to identify the missing color or number and respond by pressing the corresponding pedal. In the difficulty level 1 represented in the upper panel, number and color blocks present solid colored squares or numbers displayed in white ink on a black background. In the difficulty level 2 represented in the lower panel, both color and number blocks include numbers displayed in different colors embedded within the individual squares. During each trial, one of the four possible colors and numbers is omitted. Participants are instructed to identify the missing color or number and to respond by pressing the corresponding pedal.

The following 7 difficulty levels are implemented for the Concentration Grid Task.

Level 1 – The grid size is 5x5. The stimuli in color blocks are colored squares and the stimuli in number blocks are numbers displayed in white ink on a black background. The number of trials per block is 15. Stimulus duration is 2 s and ISI is 1 s.

Level 2 – In this level, difficulty is increased by changing the stimuli to colored numbers and decreasing stimulus duration. The grid size is 5x5. The stimuli in both block types are numbers displayed in different colors. In the number blocks, one number is missing and in the color blocks one color is missing. The number of trials per block is 15. The stimulus duration is 1.5 s and ISI is 1 s.

Level 3 – In this level, difficulty is increased by decreasing the ISI and thereby speeding up the task. The grid size is 5x5. The stimuli in both blocks are numbers displayed in different colors. In the number blocks one number is missing and in the color blocks one color is missing. The number of trials per block is 15. The stimulus duration is 1.5 s and the ISI is 0.75 s.

Level 4 – In this level, difficulty is increased by increasing the number of trials per block thereby requiring higher levels of sustained attention. The grid size is 5x5. The stimuli in both blocks are

numbers displayed in different colors. In the number blocks, one number is missing, and, in the color blocks, one color is missing. The number of trials per block is 20. The stimulus duration is 1.5 s and the ISI is 0.75 s.

Level 5 – In this level, difficulty is increased by increasing the grid size. The grid size is 7x5. The stimuli in both blocks are numbers displayed in different colors. In the number blocks, one number is missing, and, in the color blocks, one color is missing. The number of trials per block is 20. The stimulus duration is 1.5 s and the ISI is 0.75 s.

Level 6 – In this level, difficulty is increased by increasing the grid size and by decreasing stimulus duration. The grid size is 9x5. The stimuli in both blocks are numbers displayed in different colors. In the number blocks, one number is missing, and, in the color blocks, one color is missing. The number of trials per block is 20. The stimulus duration is 1.25 s and the ISI is 0.75 s.

Level 7 – In this level, difficulty is increased by increasing the number of trials per block thereby increasing sustained attention requirements. The grid size is 9x5. The stimuli in both blocks are numbers displayed in different colors. In the number blocks, one number is missing, and, in the color blocks, one color is missing. The number of trials per block is 25. The stimulus duration is 1.25 s and the ISI is 0.75 s.

4. Working Memory Training Task

In the Working Memory Training Task, each trial starts with a color or number sequence, followed by a waiting period signaled with the Portuguese word for WAIT. After a few seconds the Portuguese word for REPEAT is presented (Suppl. Fig. 8). The participants are instructed to

reproduce the memorized sequence upon the REPEAT sign by stepping on the corresponding pedals in the right order. This task lasts 5 min and is divided into smaller blocks of approximately 15s to 20s.

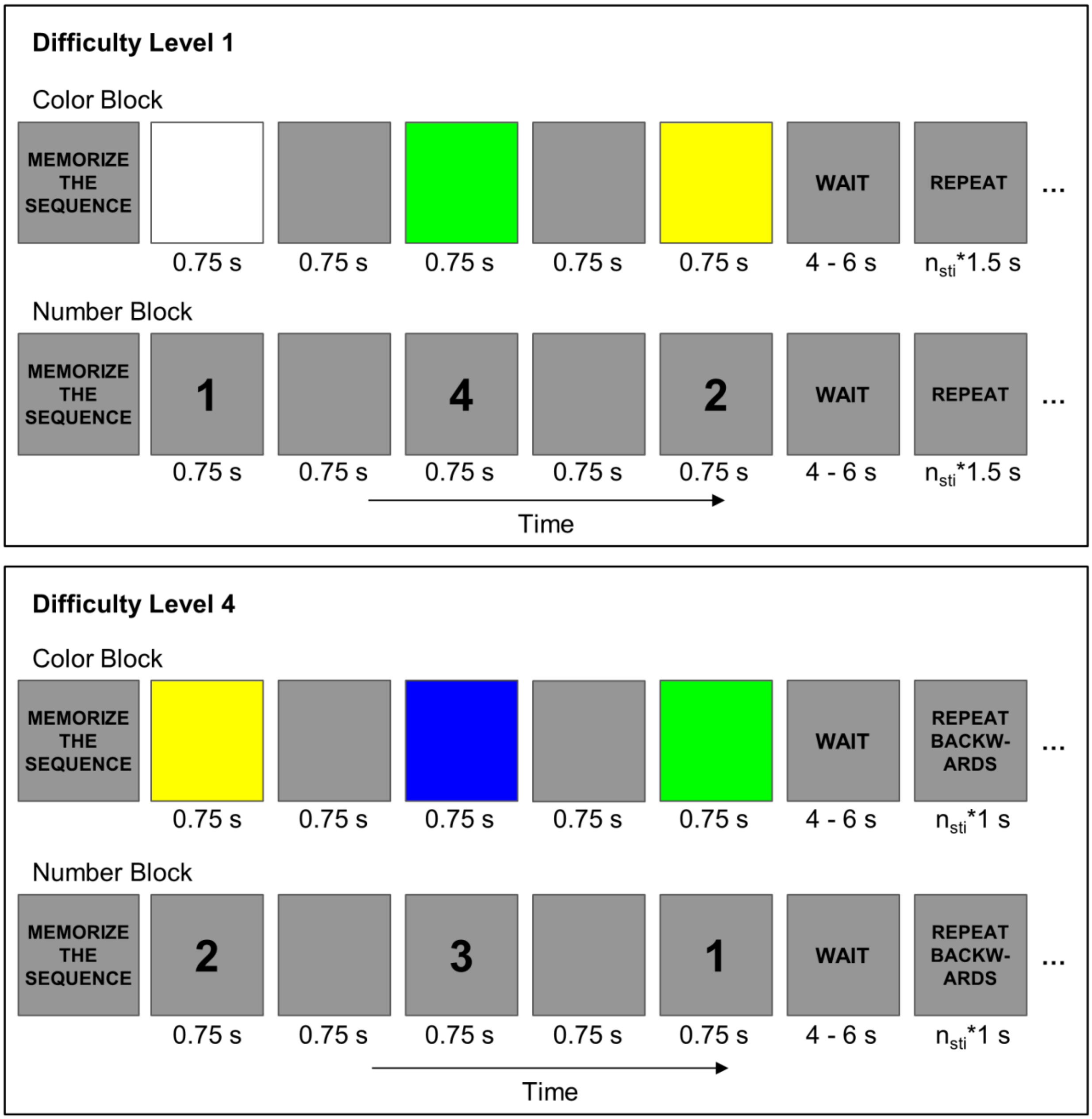

Supplementary Figure 8. Schematic representation of the structure of the Working Memory Training Task (not to scale). Each trial starts with an instructions panel alerting the participant to memorize the following

sequence. This panel is followed by a color or number sequence, followed by a waiting period signaled with the Portuguese word for WAIT. After a few seconds the Portuguese word for REPEAT is presented. The participants are instructed to reproduce the memorized sequence upon the REPEAT sign by stepping on the corresponding pedals in the right order. In the upper panel, a trial structure from the difficulty level 1 is represented. In this level, participants are instructed to reproduce the sequence from memory in the same order presented. In the lower panel, a trial structure from the difficulty level 4 is represented. In this level, participants are instructed to reproduce the sequence backwards.

The following 8 difficulty levels are implemented for the Working Memory Training Task.

Level 1 – A total of 10 number sequences and 10 color sequences are presented, in alternating order, for memorization. Each sequence contains between 2 and 4 items. The length of 6 sequences is set as 2, the length of 8 sequences is set as 3, and the length of 6 sequences is set as 4. The waiting time is defined randomly between 4 and 6 s. The time allowed for reproducing the sequence is 1.5 s times the number of items in the sequence.

Level 2 – In this level, the length of the sequences is increased. A total of 7 number sequences and 7 color sequences are presented, in alternating order, for memorization. Each sequence contains between 3 and 5 items. The length of 5 sequences is set as 3, the length of 6 sequences is set as 4, and the length of 3 sequences is set as 5. The waiting time is defined randomly between 4 and 6 s. The time allowed for reproducing the sequence is 1.5 s times the number of items in the sequence.

Level 3 - In this level, the length of the sequences is increased and the time allowed for reproducing the sequence is shortened. Each sequence contains between 4 and 5 items. A total of 8 number sequences and 8 color sequences are presented, in alternating order, for memorization. The length of 7 sequences is set as 4 and the length of 9 sequences is set as 5.

The waiting time is defined randomly between 4 and 6 s. The time allowed for reproducing the sequence is 1 s times the number of items in the sequence.

Level 4 - In this level, the participants are instructed to reproduce the sequences in reverse order. Each sequence contains between 3 and 4 items. A total of 8 number sequences and 8 color sequences are presented, in alternating order, for memorization. The length of 8 sequences is set as 3 and the length of 9 sequences is set as 4. The waiting time is defined randomly between 4 and 6 s. The time allowed for reproducing the sequence is 1 s times the number of items in the sequence.

Level 5 - In this level, the length of the sequences is increased. Participants are instructed to reproduce the sequences in reverse order. Each sequence contains between 4 and 5 items, and sequence lengths vary across trials. A total of 9 number sequences and 9 color sequences are presented, in alternating order, for memorization. The length of 13 sequences is set as 4 and the length of 5 sequences is set as 5. The waiting time is defined randomly between 4 and 6 s. The time allowed for reproducing the sequence is 1 s times the number of items in the sequence.

Level 6 - In this level, the number of longer sequences is increased. Participants are instructed to reproduce the sequences in reverse order. Each sequence contains between 4 and 5 items. A total of 8 number sequences and 8 color sequences are presented, in alternating order, for memorization. The length of 8 sequences is set as 4 and the length of 8 sequences is set as 5. The waiting time is defined randomly between 4 and 6 s. The time allowed for reproducing the sequence is 1 s times the number of items in the sequence.

Level 7 - In this level, the number of longer sequences is further increased. Participants are instructed to reproduce the sequences in reverse order. Each sequence contains between 4 and

5 items. A total of 8 number sequences and 8 color sequences are presented, in alternating order, for memorization. The length of 6 sequences is set as 4 and the length of 10 sequences is set as 5. The waiting time is defined randomly between 4 and 6 s. The time allowed for reproducing the sequence is 1 s times the number of items in the sequence.

Level 8 - In this level, the length of sequences and the number of longer sequences. Participants are instructed to reproduce the sequences in reverse order. Each sequence contains between 5 and 6 items, and sequence lengths vary across trials. A total of 8 number sequences and 8 color sequences are presented, in alternating order, for memorization. The length of 12 sequences is set as 5 and the length of 4 sequences is set as 6. The waiting time is defined randomly between 4 and 6 s. The time allowed for reproducing the sequence is 1 s times the number of items in the sequence.

5. Decision Making Training Task - Numerosity Discrimination

This task is a numerosity discrimination task. In each trial, a grey screen is divided into two equal halves by a vertical line. The participants are presented with different numbers of black circles on each side of the screen and are instructed to indicate, as quickly and as accurately as possible without counting the circles, the side that contains the larger number of circles by pressing on the two outer pedals that correspond to the left and the right sides of the screen. The side containing the larger numerosity is randomly assigned from trial to trial. To increase the participant's motivation and facilitate learning through error processing, feedback is provided after each stimulus as a green tick mark overlaid over the side containing the larger number of circles (Suppl. Fig. 9). The feedback signal is on the screen for 1s followed by a blank screen with duration 0.5s

before the start of the next trial. Participants' responses are recorded until the feedback appears on screen. This task lasts around 5 min and contains 90 trials. Every block of 10 trials contains one easy trial where one side contains between 3 and 6 circles and the other a number of circles that is 5 times that value. This easy trial condition aimed at maintaining the participants' motivation throughout the task and served as a positive control to assess participants compliance with the instructions. The other trials are constructed by using the following ratios between stimulus pairs: 4:3 and 10:9. The individual numerosities are rounded to the closest whole number.

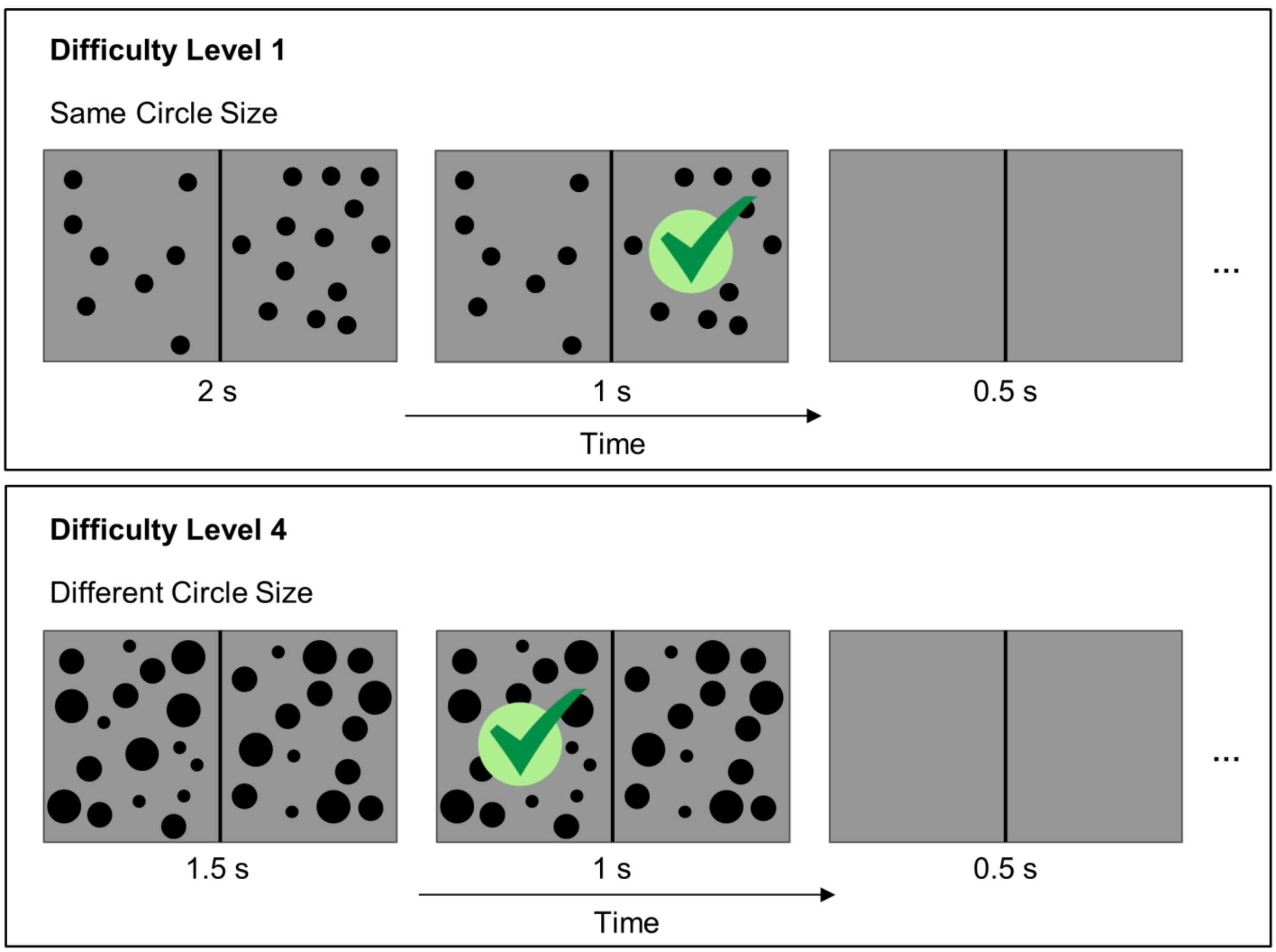

Supplementary Figure 9. Schematic representation of the structure of the Decision-Making Training Task (not to scale). In this task, participants are presented with different numbers of circles on each side of the screen and are instructed to indicate the side that contains the larger number of circles. In the difficulty level

1, represented in the upper panel, the circles are all the same size. In the difficulty level 4 represented in the lower panel, the circles have different sizes and stimulus duration is decreased increasing task difficulty.

The following 6 difficulty levels are implemented for the Decision-Making Training Task.

Level 1 – All circles have equal sizes. In each block of 10 trials, 4 trials have a ratio of 4:3 and 5 trials have a ratio of 10:9. The numerosity of the side with the lower number of circles is between 6 and 24. The stimulus duration is 2 s.

Level 2 – In this level, difficulty is increased by assigning variable sizes to the circles. Circles have randomly defined sizes. In each block of 10 trials, 4 trials have a ratio of 4:3 and 5 trials have a ratio of 10:9. The numerosity of the side with the lower number of circles is between 6 and 24. The stimulus duration is 2 s.

Level 3 – In this level, difficulty is increased by decreasing the stimulus duration. Circles have randomly defined sizes. In each block of 10 trials, 4 trials have a ratio of 4:3 and 5 trials have a ratio of 10:9. The numerosity of the side with the lower number of circles is between 6 and 24. The stimulus duration is 1.5 s.

Level 4 - In this level, difficulty is increased by including stimuli with more circles. Circles have randomly defined sizes. In each block of 10 trials, 4 trials have a ratio of 4:3 and 5 trials have a ratio of 10:9. The numerosity of the side with the lower number of circles is between 6 and 42. The stimulus duration is 1.5 s.

Level 5 - In this level, difficulty is increased by including a higher percentage of trials with a smaller numerosity ratio. Circles have randomly defined sizes. In each block of 10 trials, 3 trials have a

ratio of 4:3 and 6 trials have a ratio of 10:9. The numerosity of the side with the lower number of circles is between 6 and 42. The stimulus duration is 1.5 s.

Level 6 – In this level, difficulty is increased by decreasing the stimulus duration. Circles have randomly defined sizes. In each block of 10 trials, 3 trials have a ratio of 4:3 and 6 trials have a ratio of 10:9. The numerosity of the side with the lower number of circles is between 6 and 42. The stimulus duration is 1.25 s.

REFERENCES

1. Kleiner M, Brainard, D, Pelli, Denis, Ingling, A, Murray, R, Broussard, C. What's new in Psychtoolbox-3? *Perception*. 2007;36(14):1-16.
2. Brainard DH. The Psychophysics Toolbox. *Spat Vis*. 1997;10(4):433-436.
3. Harris D.V., Harris B.L. *The Athlete's Guide to Sports Psychology: Mental Skills for Physical People. Volume 1. Human Kinetics;* Vol 1. Champaign, IL, USA: Human Kinetics; 1984.
4. Greenlees I, Thelwell R, Holder T. Examining the efficacy of the concentration grid exercise as a concentration enhancement exercise. *Psychology of Sport and Exercise*. 2006;7(1):29-39. doi:10.1016/j.psychsport.2005.02.001
